# Supplementary figures and images for: Lower Cognitive Set Shifting Ability Is Associated With Stiffer Balance Recovery Behavior and Larger Perturbation-Evoked Cortical Responses in Older Adults
Source: Front Aging Neurosci. 2021 Dec 6;13:742243. doi: 10.3389/fnagi.2021.742243 (PMC8685437; doi:10.3389/fnagi.2021.742243)

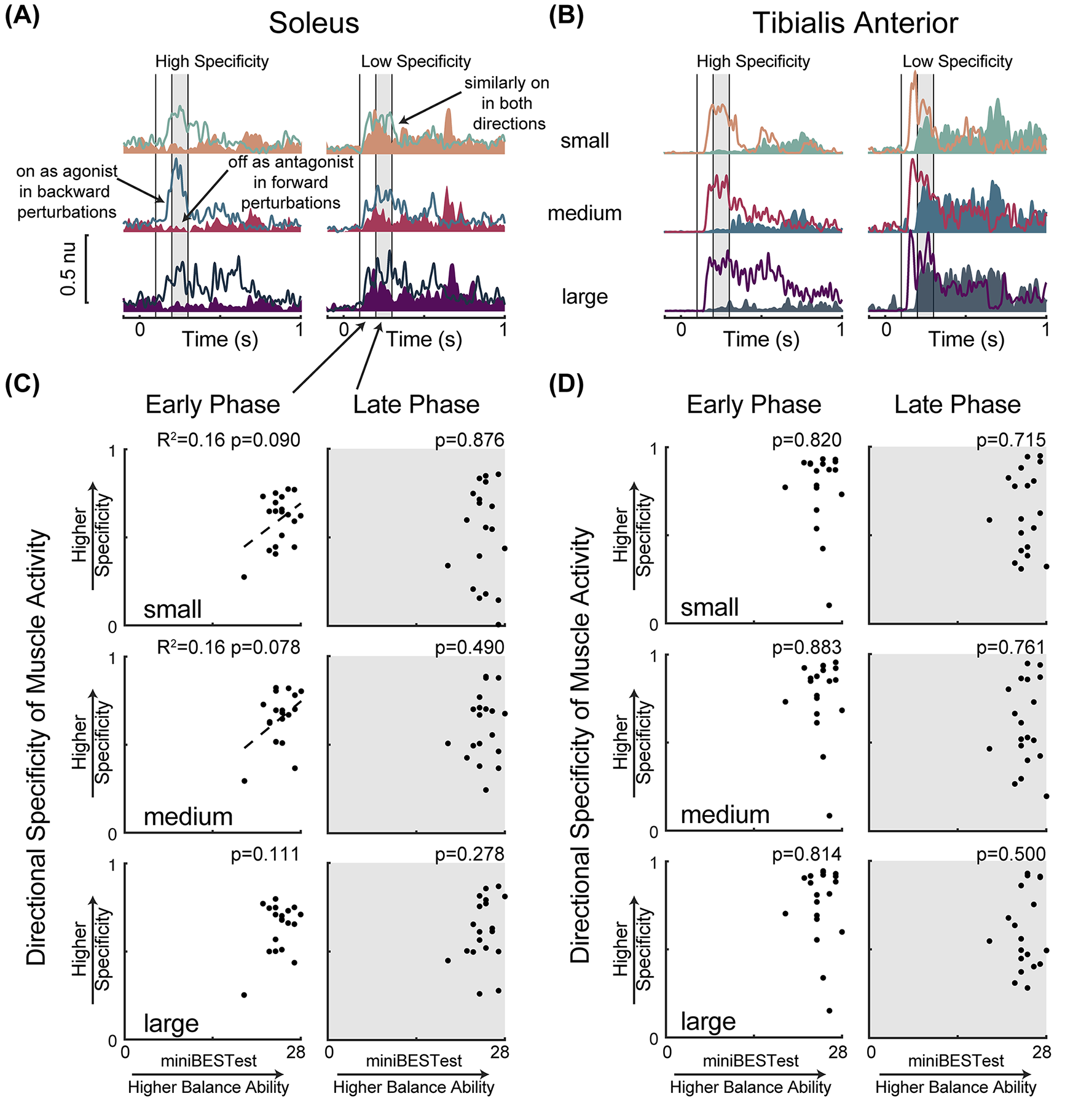

Supplement: Supplementary Figure 1 — No significant associations were observed between clinical balance ability and directional specificity of muscle activity. Condition-averaged muscle activity is shown for (A) soleus and (B) tibialis anterior muscles for example participants with higher or lower directional specificity scores. Muscle activity evoked by forward movements of the floor (resulting in backward leaning posture) is represented in magentas and activity evoked by backward movements of the floor is represented in blues, with darker colors for larger perturbations. Antagonist activity (i.e., soleus activity in forward perturbations and tibialis anterior activity in backward perturbations) is shaded for clarity. Vertical lines at 100 ms, 200 ms, and 300 ms mark the bounds of the time bins of interest. The later (200–300 ms) time bin is shaded in all panels. Directional specificity is plotted against miniBESTest scores for the (C) soleus muscle and (D) tibialis anterior muscle in each perturbation magnitude for early (100–200 ms) and late (200–300 ms) time bins. [file Image_1.TIF]

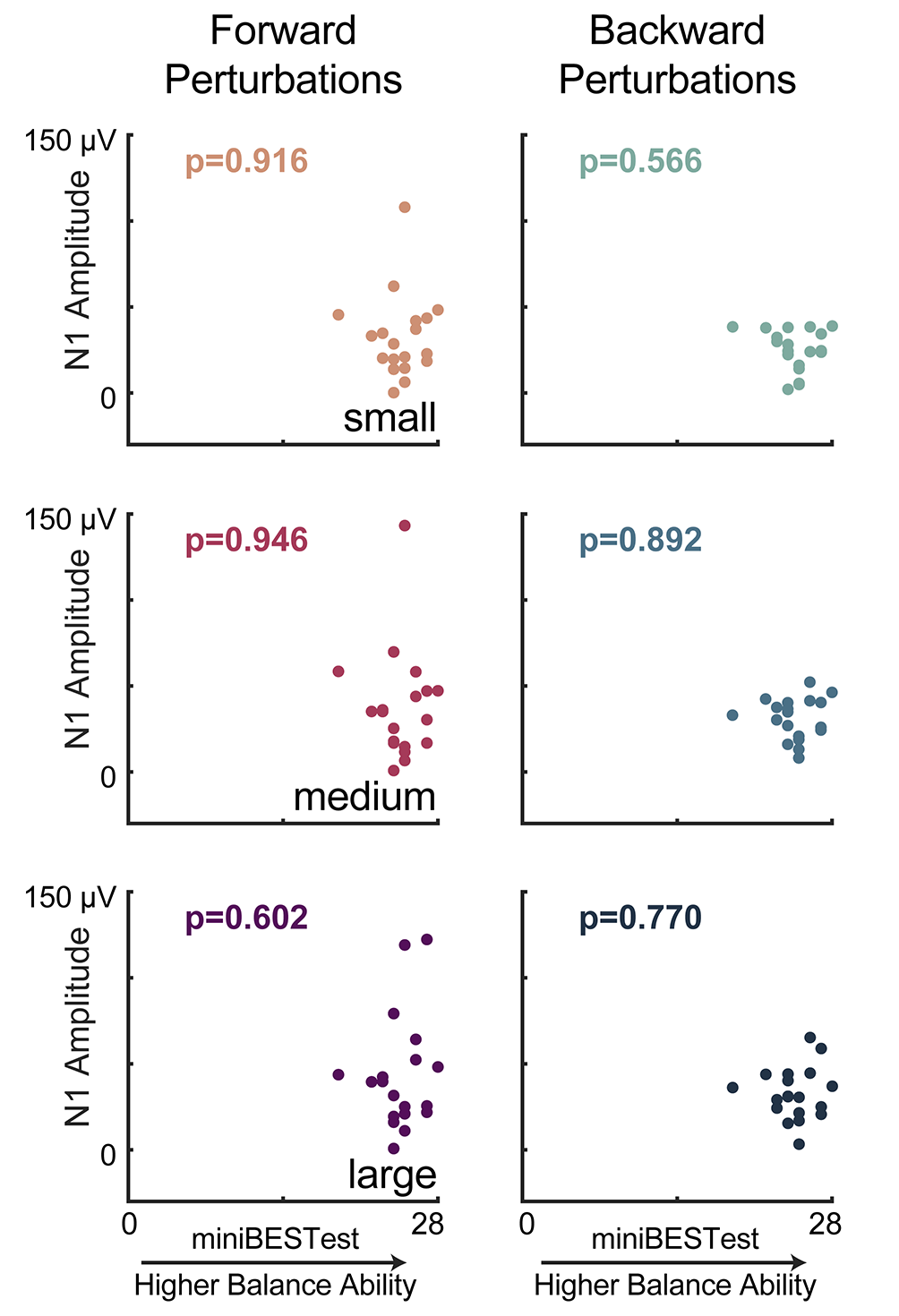

Supplement: Supplementary Figure 2 — Perturbation-evoked cortical N1 responses were not associated with clinical balance ability. Cortical N1 response amplitudes are plotted against miniBESTest scores in each of the perturbation conditions. Forward movements of the floor (resulting in backward leaning posture) are represented in magentas and backward movements of the floor are represented in blues, with darker colors for larger perturbations. [file Image_2.TIF]
